# Nanostructural Characterization of Luminescent Polyvinyl Alcohol/Graphene Quantum Dots Nanocomposite Films

**DOI:** 10.3390/nano14010005

**Published:** 2023-12-19

**Authors:** Dhanumalayan Elumalai, Beatriz Rodríguez, Ganna Kovtun, Pedro Hidalgo, Bianchi Méndez, Shaik Kaleemulla, Girish M. Joshi, M. Teresa Cuberes

**Affiliations:** 1 Department of Applied Mechanics and Project Engineering, Mining and Industrial Engineering School of Almaden, University of Castilla-La Mancha, 13400 Almadén, Spain; dhanumalayan.e2016@vitstudent.ac.in (D.E.); ganna.kovtun@uclm.es (G.K.); gm.joshi@marj.ictmumbai.edu.in (G.M.J.); 2 Thin Films Laboratory, Center for Functional Materials, Vellore Institute of Technology, Vellore 632014, Tamilnadu, India; kaleem@vit.ac.in; 3 Department of Physics of Materials, University Complutense of Madrid, 28040 Madrid, Spain; berodr03@ucm.es (B.R.); phidalgo@ucm.es (P.H.); bianchi@ucm.es (B.M.); 4 Institute of Magnetism NAS of Ukraine and MES of Ukraine, 03142 Kyiv, Ukraine; 5 Department of Engineering Physics and Engineering Materials, Institute of Chemical Technology Mumbai, Marathwada Campus, Jalna 431203, Maharashtra, India

**Keywords:** polyvinyl alcohol, graphene quantum dots, atomic force microscopy, ultrasonic force microscopy, friction force microscopy, photoluminescence, X-ray diffraction, infrared spectroscopy, thermogravimetry, differential thermal analysis

## Abstract

This study focuses on the fabrication of polymer nanocomposite films using polyvinyl alcohol (PVA)/graphene quantum dots (GQDs). We investigate the relationship between the structural, thermal, and nanoscale morphological properties of these films and their photoluminescent response. Although according to X-ray diffraction (XRD), Fourier-transform infrared spectroscopy (FT-IR), and differential thermal analysis (DTA), the incorporation of GQDs does not significantly affect the percentage crystallinity of the PVA matrix, for a range of added GQD concentrations, atomic force microscopy (AFM) showed the formation of islands with apparent crystalline morphology on the surface of the PVA/GQD films. This observation suggests that GQDs presumably act as nucleating agents for island growth. The incorporation of GQDs also led to the formation of characteristic surface pores with increased stiffness and frictional contrast, as indicated by ultrasonic force microscopy (UFM) and frictional force microscopy (FFM) data. The photoluminescence (PL) spectra of the films were found to depend both on the amount of GQDs incorporated and on the film morphology. For GQD loads >1.2%wt, a GQD-related band was observed at ~1650 cm^−1^ in FT-IR, along with an increase in the PL band at lower energy. For a load of ~2%wt GQDs, the surface morphology was characterized by extended cluster aggregates with lower stiffness and friction than the surrounding matrix, and the PL signal decreased.

## 1. Introduction

Graphene quantum dots (GQDs) are intriguing emerging materials among carbon allotropes, as they possess a nonzero band gap and present size-dependent properties. The latter are attributed to quantum confinement and edge effects. One of the main characteristics of GQDs is the luminescence property, emerging because of electron confinement in all the special dimensions [1,2,3]. Tunable properties of GQDs are achieved through synthesis processes by controlling their size and tailoring their emission characteristics. The synthesis routes of GQDs include several procedures, such as chemical exfoliation, lithography, and the hydrothermal method, where coal, graphene, graphene oxide and reduced graphene oxide are used as the source material. The aqueous dispersion of GQDs is enabled due to the oxygen functional groups present at their edges. The functionalization of GQDs also showed improved properties in tailoring both electrical and optical properties. GQDs have a broad range of applications that extend across various sectors, including their use in light emitting diodes (LEDs), LED displays, photovoltaic devices, as well as in the fields of bio-imaging, bio-sensors, and electrochemical sensors, among other areas [4,5,6,7]. Liquid-suspended GQDs glow under UV light and have UV excitation and PL emission depending on their size and properties [4]. By introducing GQDs in a polymer system, one can prepare GQD-based highly stable polymer composites with superior electrical and luminescent responses [8,9].

Polyvinyl alcohol (PVA) is a unique synthetic polymer obtained by partial or complete hydrolysis of polyvinyl acetate (PVAc) by replacing the acetate group (CH_3_COO) with the hydroxyl (–OH) group. The chemical structure of PVA favors the formation of hydrogen bonds and gives it a hydrophilic character. PVA is well known for its facility to form stable films, superior optical transparency, and high solubility in water. PVA has been highly researched for its ability as a biocompatible carrier for drug delivery applications in clinical studies, and for its biodegradable property [10,11]. For decades, various fillers have been incorporated into PVA films and their structure-property relationship has been investigated. GQDs have also been used as fillers in PVA matrices and the enhancement of their luminescence properties has been explored [2,12,13,14,15,16,17,18,19,20,21,22,23,24]. 

In this work, we prepared PVA/GQD nanocomposites with varying GQD content from 0.4 up to 2.0%wt, and we carried out a thorough study of their structural, thermal, and morphological properties on the nanometer scale using X-ray diffraction (XRD), Fourier-transform infrared spectroscopy (FT-IR), thermogravimetry (TGA), differential thermal analysis (DTA), and atomic force microscopy (AFM)-based techniques, including ultrasonic force microscopy (UFM) and lateral force microscopy (LFM). The results are correlated with the photoluminescent response of the nanocomposite films when excited with a 325 nm laser source. The photoluminescence (PL) spectra of the films were found to depend on both the amount of GQD incorporated and the film surface morphology, even in those cases where neither XRD, FT-IR, nor DTA revealed significant structural changes. 

We present here an in-depth characterization of the nanostructure of PVA/GQD films using scanning probe microscopy (SPM) techniques. In addition, the SPM data have been significantly related to photoluminescence (PL) emission results, which is of extreme relevance. Although the photoluminescence of GQD fillers within PVA matrices has already garnered significant interest [2,12,13,14,15,16,17,18,19,20,21,22,23,24], a thorough investigation of the nanostructural modifications occurring at the surface of PVA/GQD nanocomposites is still lacking. Understanding and manipulating the nanostructure of these films is crucial for tailoring their properties, constituting a fundamental step toward harnessing the full potential of these materials for practical use. 

## 2. Materials and Methods

### 2.1. Preparation of the PVA/GQD Nanocomposite Films

**Figure 1:**
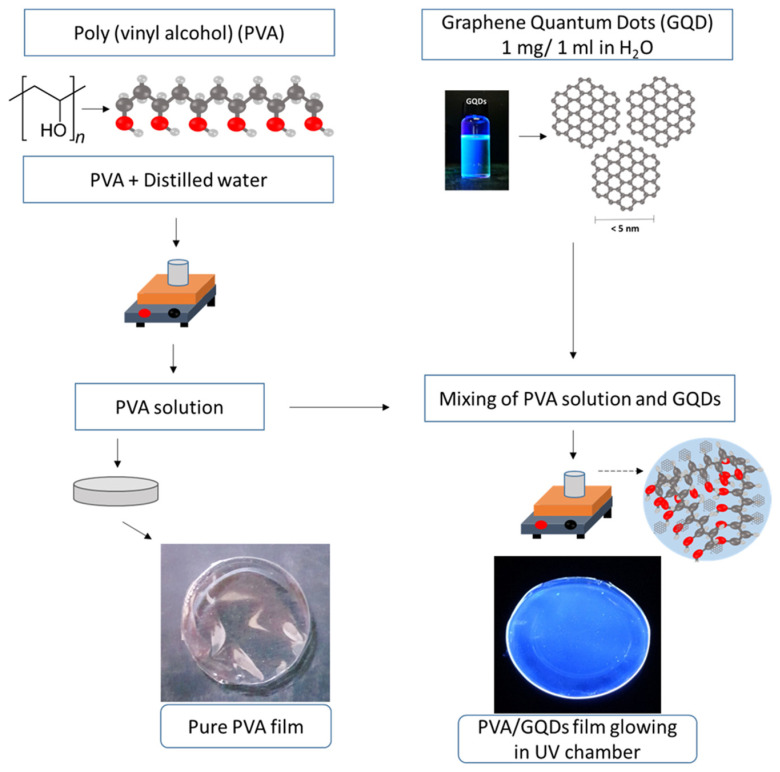
Schematics of the pure PVA and PVA/GQD nanocomposite films preparation procedure.

Polyvinyl alcohol (PVA) in granular form (MW 31,000–50,000, 98–99% hydrolyzed) and graphene quantum dots < 5 nm in diameter, with maximum emission between 435 and 450 nm, in aqueous solution with a concentration of 1 mg/mL, were purchased from Merck, Darmstadt, Germany [4]. The preparation of the PVA/GQD nanocomposite films was carried out at ambient conditions (temperature ~30 °C, relative humidity ~30%RH). First, a 6%wt stock solution of pure PVA was prepared by dissolving PVA granules in distilled water under constant stirring at 900 rpm and 90 °C until complete dissolution. Next, various amounts of GQD solution were added to the PVA stock solution to obtain mixed solutions with 0.4–2.0 weight percentage of GQD NPs relative to PVA (denoted as %wt GQD), and the resulting mixtures were further stirred at 900 rpm and 60 °C for ~5 h. Finally, the mixtures were poured into Petri dishes, labelled appropriately, and kept at room temperature for the evaporation of excess water. In about 36 h, PVA/GQD films, a few microns thick, could be easily peeled off from the containers. Figure 1 illustrates the schematics of the pure PVA and the PVA/GQD nanocomposite films preparation procedure. When introducing the PVA/GQD nanocomposites into a UV chamber, an intense blue emission could be observed even for those films with the lowest GQD concentrations (see Figure 1). 

### 2.2. X-ray Diffraction

X-ray diffraction measurements were performed using the Philips X’Pert MPD equipment (Eindhoven, Holland), employing CuK α radiation (1.54056 Å) at 40 KV and 40 mA. It incorporates 0.04 rad soller slits for both incident and diffracted beams, an automatic 12.5 mm programmable divergence slit, and a Xe gas-sealed proportional detector. Data were collected in an angular range between 3° and 50° (2θ) with a step size of 0.02° and a counting time of 0.70 s per step. The data analysis was carried out with OriginPro 2022b software.

### 2.3. Fourier-Transformed Infrared Spectroscopy

FTIR spectra (4 cm^−1^ resolution, wavenumber range 500–4000 cm^−1^) were recorded using a Shimadzu IRPrestige-21 spectrometer (Tokyo, Japan), using the ATR method. Small pieces of the PVA/GQD nanocomposites hybrid films (≈10 µm thick) were cut and placed in the instrument sample holder. The data were acquired and analyzed using Shimadzu IR solution 1.21 software (Tokyo, Japan).

### 2.4. Thermogravimetry and Differential Thermal Analysis

Thermogravimetry and differential thermal analysis were carried out using a SETARAM model TG/DTA92 instrument (Caluire-et-Cuire, France) over the temperature range of 30–500 °C with a heating rate of 5 °C/min in a Pt crucible in an air atmosphere. 

### 2.5. Scanning Probe Microscopy 

Contact-mode atomic force microscopy (AFM), lateral force microscopy (LFM) and ultrasonic force microscopy (UFM) were performed using a NANOTEC (Madrid, Spain) instrument. The modification of the AFM equipment for the incorporation of UFM facilities is described in [25]. For UFM, ultrasonic frequencies of ~3.8 MHz and modulation frequencies of 2.4 KHz were applied from a piezoelectric element placed under the sample. Typically, Olympus Silicon Nitride cantilevers with a nominal spring constant of 0.06 N/m and a nominal tip radius of 20 nm were used. The measurements were performed in air, at ambient conditions. Data analysis was performed with WSxM 4.0 Beta 9.3 software (Madrid, Spain). 

### 2.6. Photoluminescence

Photoluminescence measurements were carried out at room temperature on a Horiba Jobin-Yvon LabRam Hr800 (Horiba, Kyoto, Japan) using a continuous wave He-Cd laser (λ = 325 nm). Different neutral filters were used to attenuate the total laser intensity, when necessary, diminishing it from the nominal 5 mW. The laser was focused onto the sample surface using a 40× objective (numerical aperture = 0.5, Thorlabs LMU-40X-NUV, Newton, NJ, USA), which led to a laser spot diameter of around 1 µm for the UV laser. The scattered light was collected with the same objective, dispersed with a grating of 600 L/mm, and finally acquired with an air-cooled CCD detector Synapse.

## 3. Results and Discussion

**Figure 2:**
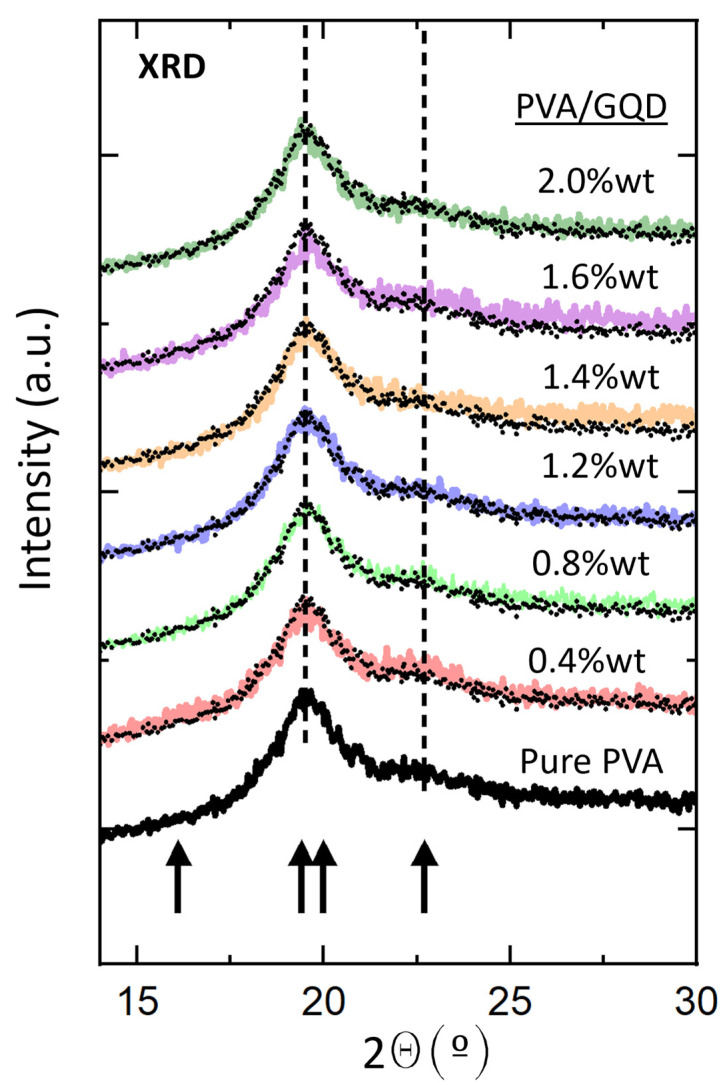
Normalized XRD patterns of the pure PVA and PVA/GQDs composite films. The dotted curve reproduces the diffractogram of the pure PVA film that has been superimposed on each of the diffractograms of the PVA/GQD nanocomposites with different GQD content, depicted in different colors for ease of comparison. The arrows indicate the position of expected maxima in crystalline PVA [26].

PVA exhibits semicrystalline properties due to inter- and intra-molecular hydrogen bonding (O–H) that provides the structural order of PVA chains [26,27]. Figure 2 shows the normalized XRD patterns of the pure PVA and PVA/GQDs composite films. The lowest black curve in Figure 2 corresponds to the diffractogram of the pure PVA film. Within the considered angular range, four crystalline maxima at 2θ≈16.1° indexed as (001), $2\theta \approx 19.4{}^{\circ}\;\left(10\bar{1}\right)$, $2\theta \approx 20.0{}^{\circ}\;\left(101\right),\;\mathrm{and}\;2\theta \approx 22.7{}^{\circ}\;\left(200\right)$ should be distinguished. The positions are indicated by arrows in Figure 2. Nevertheless, in our case, peaks at 19.4° and 20.0° were not resolved.

Considering a peak at 2θ≈19.5° of FWHM of 2.1° and applying the Debye-Scherrer equation,

$$D=\frac{K\;\lambda }{FWHM\;cos\;\theta }$$

where *D* is the crystalline size, *K* the Scherrer constant (0.98), and λ the wavelength (0.154 nm), a size of D≈ 4.2 nm is estimated for the PVA crystallites. This size is approximately the same as that of the GQD (nominal size < 5 nm). 

The interplanar spacing may be estimated as d≈4.5 Å, taking into account Bragg’s equation,

$$n\lambda =2d\left(sin\;\theta \right)$$

where *n* is the order of reflection. 

The % of crystallinity was calculated as ≈18% by considering the ratio between the area of crystalline peaks and the total area in the XRD diffractogram. 

The normalized diffractograms of the PVA/GQD films with different GQD loadings correspond to the curves in different colors in Figure 2. In each case, the diffractogram of the pure PVA film has been superimposed (black dotted curve) on the corresponding curve for ease of comparison. As can be seen in Figure 2, the shape of the diffractogram does not undergo any significant change for the PVA/GQD films with different %wt loadings of GQD. This fact indicates that neither the crystallinity percentage of the films nor the average size of the PVA crystalline domains should be significantly affected by the incorporation of GQD in our PVA/GQD nanocomposite films. Moreover, it is evident that no additional peak related to the presence of GQD is distinguished, even for the highest GQD load considered (2%wt). Previous works have reported the detection of a characteristic broad peak in the 2θ range of ~21 to 25°, arising from the diffraction in GQD (002) planes [28,29]. However, this peak originates from interlayer stacking, and its intensity is expected to be lower and the peak broader, with fewer graphene layers forming the GQD. According to [4], our purchased GQDs have a topographic height of 1–2.0 nm. In any case, the PVA diffraction peak at 23° (indicated with an arrow in Figure 2) would overlap with a diffraction signal originating from the embedded GQDs in the same 2θ range.

**Figure 3:**
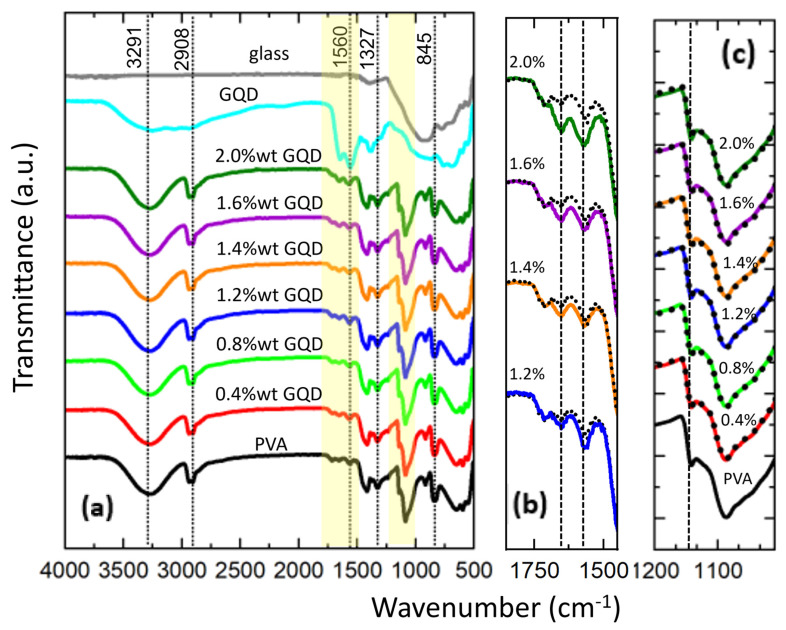
Normalized FT-IR spectra of the pure PVA and PVA/GQD composite films. (**a**) The FT-IR spectrum of GQDs deposited on a glass slide (cyan curve) and of the clean glass slide (grey curve) is included. (**b**,**c**) Zoom of the regions highlighted in yellow in (**a**). The dotted black curve corresponds to the FT-IR spectrum of pure PVA which has been shifted and superimposed on each of the other PVA/GQD film’s FT-IR spectra for ease of comparison.

The chemical structure of PVA/GQD composite films has been analyzed with reference to the pure PVA film using FT-IR spectroscopy. Figure 3 shows the normalized FT-IR spectra for the pure PVA and PVA/GQD composite films. To characterize the FT-IR response of the GQDs, a special sample was prepared by depositing a droplet of the GQD solution on a glass slide and waiting for 24 h for the solvent to evaporate. The FT-IR spectra of the GQDs (on the glass slide) and the clean glass slide without deposited GQDs are also shown in Figure 3. 

In Figure 3a, the broad band in the range of 3600 cm^−1^ to 3100 cm^−1^ is attributed to –OH stretching as a result of inter- and intra-molecular hydrogen bonding of PVA. An –OH band around the same range also appears in the GQD spectrum. However, in the spectra of the PVA/GQD films, no significant modification of the -OH peak with respect to the spectrum of the pure PVA is apparent, neither in shape nor in position of the maximum. For the highest GQD loads, a slight shift of the OH band maximum to higher wavenumbers can be allocated (by ~10 cm^−1^ for the 2%wt GQD load, almost inappreciable in Figure 3a), possibly due to the incorporation of the GQDs into the PVA matrix. 

The peaks at 2935 cm^−1^ and 2908 cm^−1^ are assigned to the symmetric and asymmetric CH_2_ stretching modes, and their shape and position remain the same for the pure PVA and the different PVA/GQD composites. 

In the spectrum of GQD (cyan curve in Figure 3a), bands appear at 1650 cm^−1^ and 1560 cm^−1^; however, these bands, although weak, are also found in the pure PVA and the PVA/GQD films. In pure PVA, the band at ~1654 cm^−1^ was attributed to absorbed water [30,31]. Given the fact that the GQD sample was prepared from an aqueous solution, it is plausible that some water molecules remained attached to the GQDs after the evaporation of the solvent, giving rise to this band. In the case of GQDs, such a band has been previously assigned to the in-plane stretching vibration of the sp^2^ hybridized C=C bond [32]. According to our data, it is clear that, in our case, this peak can be associated with the presence of GQDs. The peak at 1560 cm^−1^ is attributed to C=O stretching [33]. Although our PVA was completely hydrolyzed (98 to 99%), some residual acetate groups in the PVA molecular chains contained carbonyl bonds, which may explain the weak peak observed at ~1560 cm^−1^ in pure PVA and PVA/GQD films. In addition, GQDs are expected to have attached surface carboxyl groups (–COOH), with a characteristic absorption band around 1566 cm^−1^ [32] and 1570 cm^−1^ [34].

The modification of the PVA/GQD FT-IR spectra in the spectral region from 1850 to 1450 cm^−1^ is carefully investigated in Figure 3b. There, the FT-IR spectrum of pure PVA (black dotted curve in Figure 3b) has been superimposed on the spectra of PVA/GQD with different GQD loadings. For GQD loadings less than 1.2%wt, no appreciable difference can be distinguished between the pure PVA and PVA/GQD spectral curves. However, at GQD loadings of 1.2%wt, a slight increase in the bands coincident with those of the GQDs (cyan curve in Figure 3a) is seen, and the increase becomes larger as the amount of GQDs incorporated into PVA matrix increases. 

In Figure 3a, the peaks around 1417 cm^−1^ and 1327 cm^−1^ are assigned to –OH bending (in-plane) and C–H wagging modes of PVA [35]. These peaks also remain identical in shape and position for the different PVA/GQD composites. 

Particularly interesting is the peak at 1141 cm^−1^, attributed to C–O/C–C stretching modes, which is typically used to evaluate the crystallinity of PVA using FT-IR analysis [31,36,37]. The FT-IR spectra of the pure PVA and PVA/GQD composites in the spectral region from 1200 to 1019 cm^−^^1^ are carefully investigated in Figure 3c. There, the FT-IR spectrum for pure PVA (black curve) has been vertically shifted and superimposed (black dotted curves) on the spectra of PVA/GQD with different GQD loadings. As can be seen in Figure 3c, the spectral curves do not change in shape or position as the amount of GQD incorporated into the PVA matrix increases. These results confirm the XRD observations that the percentage crystallinity of pure PVA is not affected by the GQD loading. 

Eventually, in Figure 3a, the peaks at 1089 cm^−1^, 916 cm^−1^, and 845 cm^−1^ are attributable to the C–O stretching, CH_2_ rocking mode, and C-C stretching vibrational modes, respectively [38,39], which do not undergo any modification in shape or position in the pure PVA films and PVA/GQD composites. Table 1 lists the peak assignments discussed in the FT-IR spectra of Figure 3. 

The thermal stability and thermal transition properties of the PVA/GQD samples were studied over a varying temperature range via TG/DTA analysis. 

**Figure 4:**
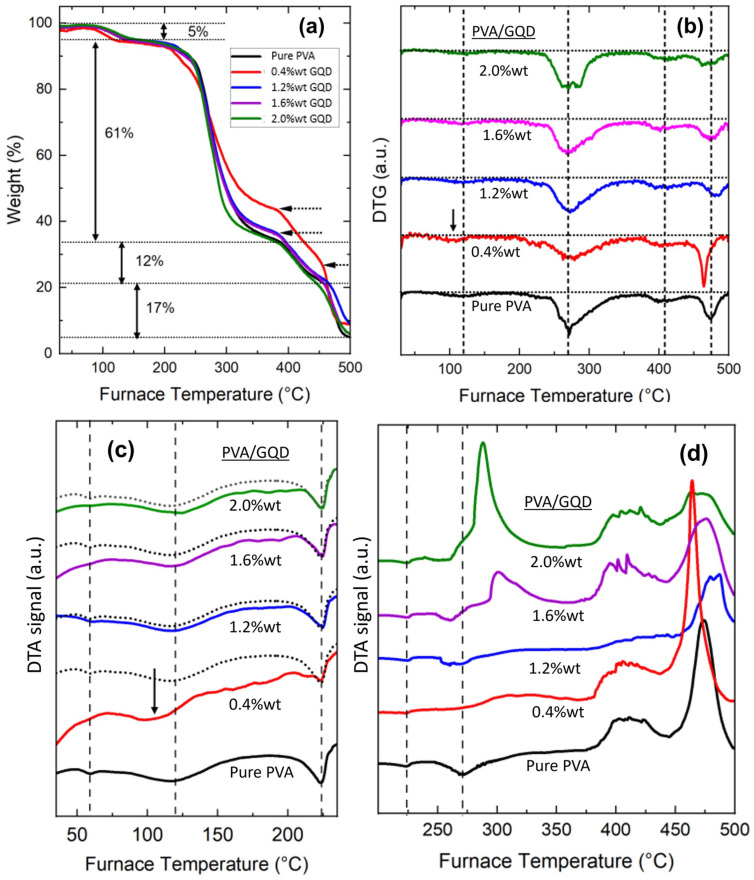
(**a**) TGA thermographs, (**b**) DTG curves and (**c**,**d**) DTA data of pure PVA and PVA/GQD composite films.

From Figure 4a,b, four degradation phases can be distinguished. The percentage weight loss of each thermal degradation phase for PVA is indicated in Figure 4a. As can be seen in Figure 4, the inclusion of GQDs in the PVA matrix influences the thermal behavior of the resulting nanocomposite film. Table 2 indicates the weight loss percentage for each degradation stage (measured from Figure 4a) and the % remaining at 500 °C. 

**Figure 5:**
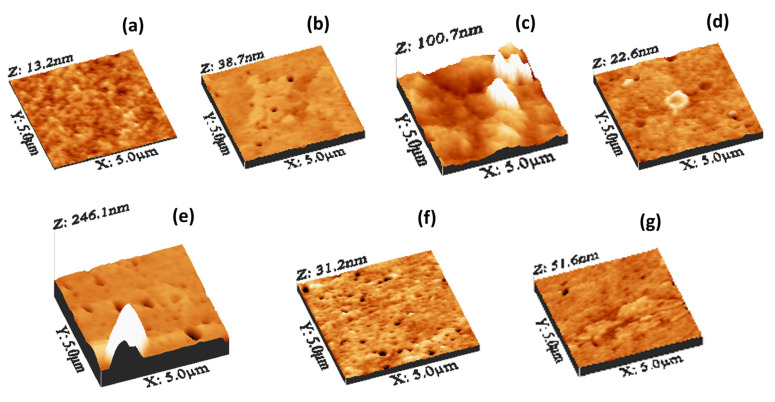
A 3D representation of AFM topographic images recorded in contact mode for (**a**) pure PVA and PVA/GQD nanocomposite films with (**b**) 0.4%wt GQD, (**c**) 0.8%wt GQD, (**d**) 1.2%wt GQD, (**e**) 1.4%wt GQD, (**f**) 1.6%wt GQD, and (**g**) 2.0%wt GQD.

**Figure 6:**
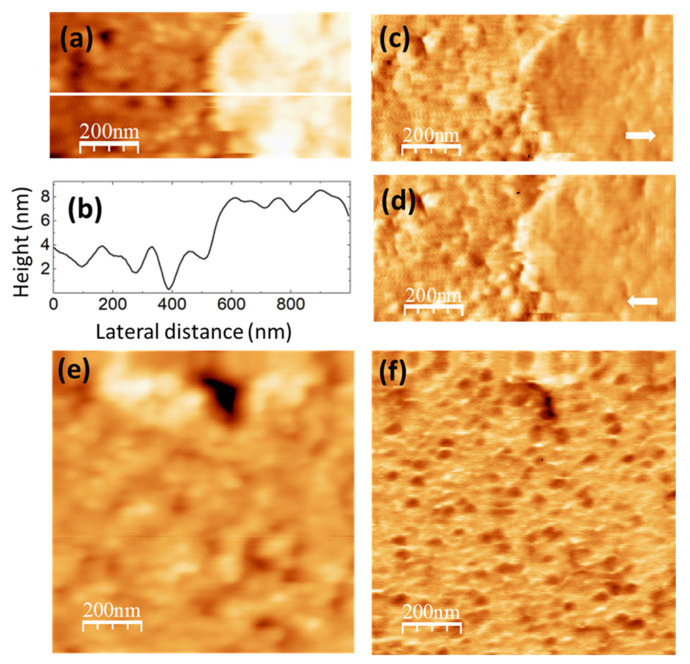
PVA/GQD film with 0.4%wt GQD. (**a**) Contact-mode AFM topography. Color-scale range: 12 nm. (**b**) Height-contour profile along the white line in (**a**). (**c**,**d**) LFM images recorded over the same surface area as (**a**), scanning from left to right (**c**) and from right to left (**d**). (**e**) Contact-mode AFM topography over a different surface area than (**a**). Color-scale range: 12 nm. (**f**) UFM image simultaneously recorded with (**e**) over the same surface area.

At the first degradation stage, a ~5% loss of weight is observed at temperatures between 70° and 160 °C in all films, which can be attributed to the evaporation of residual water within the samples [12,40]. The maximum weight loss temperature occurs at ~120 °C in all cases, except for the film with 0.4%wt GQD content, where it occurs at a temperature ~15 °C lower (see arrow in Figure 4b). In the 0.4%wt GQD samples, GQDs incorporated into the PVA matrix may modify the interactions between the PVA molecular chains, facilitating the removal of residual water from the PVA film (this point will be further considered in the discussion of the SPM data for these samples, Figure 5b and Figure 6). Figure 4c,d show the differential thermal analysis data. In Figure 4c, the dashed lines indicate the endothermic peaks corresponding to the glass transition temperature, at ~59 °C, the evaporation of the residual water at ~120 °C, and the crystalline melting point at ~224 °C of the pure PVA film [41]. The curve corresponding to pure PVA has been superimposed (black dotted curves) on the curves measured for the other PVA/GQD films. No significant modification of those points is seen for the different films, except in the case of 0.4%wt GQD, for which the evaporation of the residual water occurs at ~15 °C less than for the pure PVA film (see arrow in Figure 4c), in agreement with the observations in Figure 4a,b). Moreover, the enthalpy of fusion of the different composites—area under the endothermic peak corresponding to the melting transition—is apparently similar to that of the pure PVA film for the different PVA/GQD nanocomposites, in agreement with the XRD results indicating that the percentage of crystallinity remains the same, except perhaps for the case of 0.4%wt GQD, whose DTA curve exhibits a positive slope. 

The second degradation stage takes place at temperatures between 200 and 350 °C (Figure 4a,b) and is attributed to the disruption of the intermolecular hydrogen bonding in PVA, with partial chain-stripping elimination reactions (removal of water, with the elimination of hydroxyl side-groups) and chain-scission reactions (formation of free radicals by PVA chain breakage), leading to the formation of polyenes as a result of thermal degradation [42,43]. TGA in Figure 4a indicates again a different behavior for the 0.4%wt GQD sample in this regime. In this case, the bonding of GQDs to the PVA molecules may hinder the chain-stripping reactions, leading to the observed lower degradation rate. For films with higher amount of GQDs, the structures formed by the GQD and the PVA segments may be different, with the GQDs exerting less influence on the degradation rate of the PVA matrix. From Figure 4b, for pure PVA, the maximum weight loss temperature at which the degradation occurs in this second stage is at ~270 °C, at which an endothermic peak appears in the DTA measurements for the pure PVA film (Figure 4d). Interestingly, the DTG curves (Figure 4b) also reveal a small transition in the temperature region corresponding to the melting point, at the onset of this second degradation stage, both for the pure PVA and the PVA/GQD composites. Nevertheless, when analyzing the DTA curves at temperatures close to 270 °C (see the corresponding dashed line in Figure 4d), the response of the different composites is apparently rather different. For GQD concentrations of 1.2%wt and 1.6%wt, the endothermic peak appears shifted to lower temperatures, although the maximum loss peak in Figure 4b remains at the same position. And for the higher GQD contents, i.e., 1.6%wt and 2.0%wt GQD, the DTA reveals the occurrence of an exothermic transition at this temperature, and even more significant exothermic peaks are measured within this temperature range (see Figure 4d). The results in Figure 4d evidence that the presence of GQD alters the reactions taking place during this second stage of PVA degradation. Finally, regarding the third and fourth degradation steps, above 350 °C, reactions giving rise to exothermic peaks in TDA take place (see Figure 4d). At these stages, the occurrence of further degradation and carbonization of PVA backbone structure is expected [44].

Let us study the surface features of the PVA/GQD nanocomposite films. Figure 5 shows 3D representations of the topography measured by AFM of the pure PVA and PVA/GQD nanocomposite films prepared with different GQD concentrations. As it is apparent from Figure 5, the incorporation of GQD within the PVA matrix has a strong impact on the film topographic features. The surface of the pure PVA film (Figure 5a) is characterized by the presence of rounded, homogeneously distributed clusters, ~80 nm in diameter. The Root Mean Square (RMS) roughness in Figure 5a is 1.6 nm, the surface skewness is 0.1, and the kurtosis is 3.0. However, on the sample with 0.4%wt GQD (Figure 5b), the surface structural homogeneity has been severely disrupted. The RMS roughness is now 2.1 nm, with skewness at −2.0, and kurtosis at 21.1. Surface pores are now more clearly visible on the surface, and the former cluster structures cannot be now resolved, having been replaced by a kind of extended stratified islands. 

**Figure 7:**
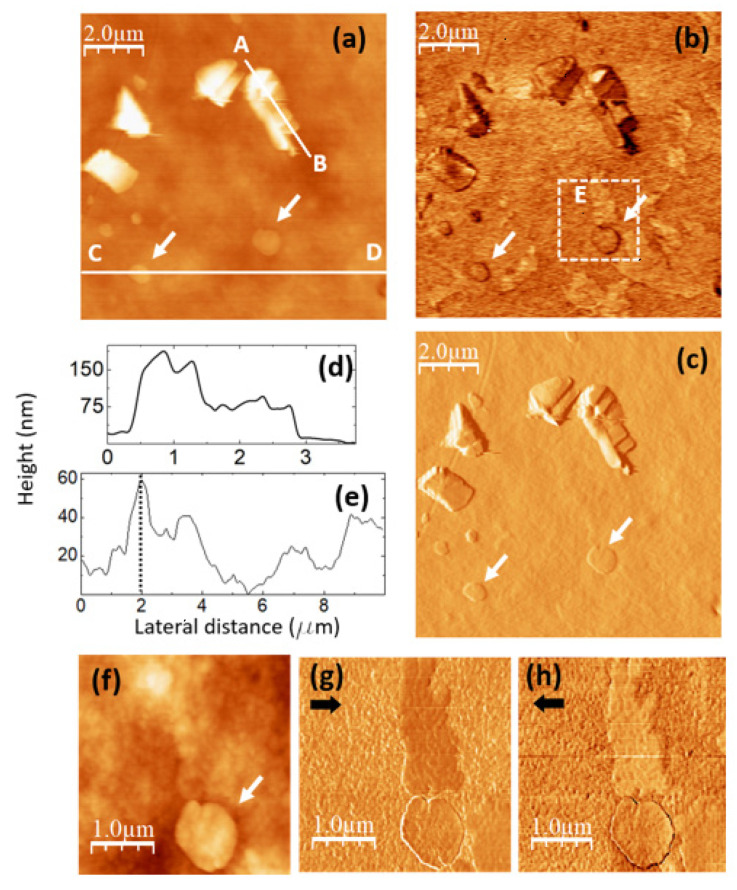
PVA/GQD film with 0.8%wt GQDs. (**a**) Contact-mode AFM image. Color-scale range: 208 nm (**b**) UFM image recorded simultaneously with (**a**), over the same surface area. (**c**) Derivative image of (**a**). (**d**) Height-contour profile recorded along the white line labelled A–B in (**a**). (**e**) Height-contour profile recorded along the lower white line labelled C–D in (**a**). (**f**) Contact-mode AFM image recorded over the area enclosed by the dashed white rectangle in (**b**). Color-scale range: 35 nm. (**g**,**h**) LFM images recorded over the same surface area as (**f**), scanning from left to right (**g**) and from right to left (**h**).

In the case of the sample with 0.8%wt GQD (Figure 5c), the changes are even more dramatic. Although XRD, FT-IR, and DTA allow us to conclude that there is no variation on the percentage crystallinity of PVA for the films with different GQD concentrations, the AFM topographic image in this film clearly reveals the presence of 3D clusters with stepped walls and facets characteristic of a crystalline morphology. We attribute these features to the formation of crystalline PVA islands, possibly with GQDs acting as a nucleating agent for 3D crystalline PVA growth on the sample surface, as will be discussed in more detail below (see the discussion related to Figure 7). In Figure 5c, the RMS roughness is 9.7 nm, the skewness is 1.8 and the kurtosis is 12.4. 

**Figure 8:**
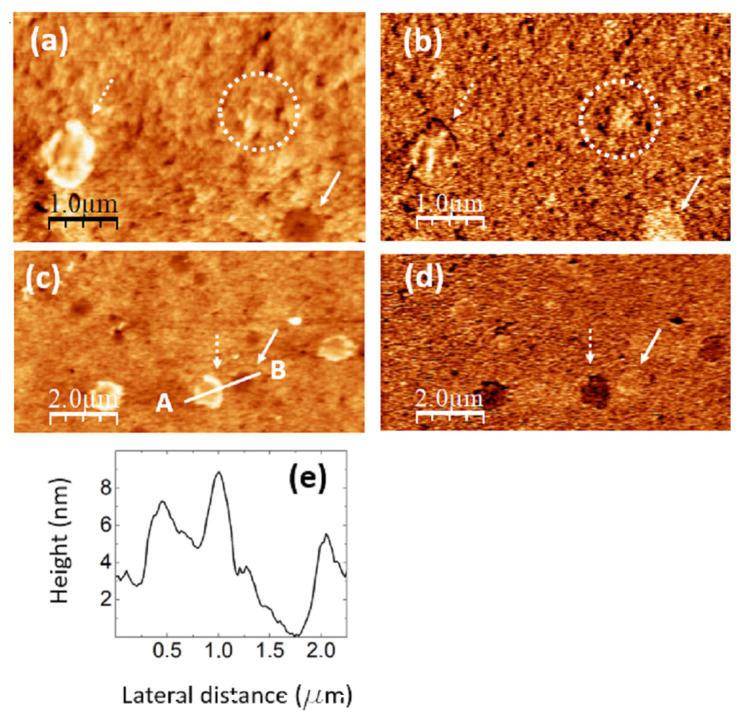
PVA/GQD film with 1.2%wt GQDs. (**a**) Contact-mode AFM topography. Color-scale range: 14 nm. (**b**) UFM image recorded simultaneously with (**a**) over the same surface area. (**c**) Contact-mode AFM topography in a different area than (**a**). Color-scale range: 14 nm. (**d**) UFM image recorded simultaneously with (**c**) over the same surface area. (**e**) Height-contour profile recorded along the lower white line in (**c**).

On the film with 1.2%wt GQDs (Figure 5d), the surface regains a flat appearance, with a surface roughness of 1.05 nm, a skewness of 0.1 and a kurtosis of 6.7. Pores and characteristic circular structures with a surrounding annular ring can be observed. Possible formation mechanisms of those structures will be discussed below, in conjunction with Figure 8. 

On the film with 1.4%wt GQDs (Figure 5e), 3D islands like those in Figure 5c were again found, together with pores similar to those in Figure 5d. Due to the presence of the 3D features and pores, the RMS roughness amounts to 12.5 nm, the skewness to 3.8 and the kurtosis to 18.4. 

For the film corresponding to 1.6%wt GQDs, in addition to (smaller) pores, small surface clusters aligned along a specific direction can be distinguished in Figure 5f. In this case, the RMS roughness is 1.4 nm, the skewness −1.3 and the kurtosis 12.17.

Eventually, for the case of 2.0%wt GQD, aligned surface clusters can be observed in Figure 5g, similar to those in Figure 5f, but now gathered to form larger aggregates. Here, the RMS roughness is 1.7 nm, the skewness is −2.3, and the kurtosis is 30.2. 

In the following, characteristic features of the different PVA/GQD films will be discussed in more detail taking advantage of the application of different AFM modes, where relevant. 

The images in Figure 6 were recorded on the PVA/GQD film with 0.4%wt GQDs. Figure 6a shows the surface topography, recorded with contact-mode AFM. Figure 6b corresponds to a height-contour profile along the white line in Figure 6a. In the lower right-hand side of Figure 6a, the presence of a terrace ~4 nm higher is apparent. On the lower terrace, rounded clusters ~80 nm in diameter similar to those on the pure PVA sample surface can be distinguished; the area is characterized by frequent “void” defects possibly consisting in displaced clusters. There are also clusters on the upper terrace, although they do not have such a well-defined rounded shape. Figure 6c,d correspond to LFM images recorded over the same surface area as Figure 6a. As it is apparent from Figure 6c,d, only a slight frictional contrast (darker in Figure 6c and brighter in Figure 6d over the same area) is noticeable at some areas over the higher terrace region, indicating a chemical homogeneity of the surface. 

Figure 6e,f correspond to contact-mode AFM topography (Figure 6e) and UFM (Figure 6f) images recorded over another surface area of the same sample. The UFM image reveals that some of the clusters topographically similar in (Figure 6e) exhibit, however, a lower UFM contrast, indicative of a lower stiffness. Such result may arise from a different conformation and packing of the macromolecular PVA chains within such clusters, resulting in a lower density. Also, it is observed that the higher topographic area at the top in Figure 6e does not lead to a notably different UFM contrast in Figure 6f, in agreement with the conclusions obtained when analyzing (Figure 6c,d).

The obtained results indicate that GQD interactions with PVA influence the conformation of the PVA chains with respect to those of pure PVA. GQDs can easily bind to a PVA chain via H-bonds through the chemical groups at their edges. According to the FT-IR data (see Figure 3a,b), our GQDs must contain groups with C=O bonds, as well as OH groups at their edges, which confers them hydrophilic nature. It is plausible that the carbon core of the GQDs promotes the formation of the void defects observed in Figure 6a,e The incorporation of GQDs into the PVA molecular chains may indeed alter the interactions between PVA molecules and/or the conformation of the PVA chains, inducing their re-arrangement into less dense clusters and/or new terraces. On the other hand, it is likely that these morphological changes lead to an increase of the available free volume for the water molecules in the film, which may lower the energy barrier for water removal, as observed by thermogravimetry (see Figure 4a–c). 

The images in Figure 7 were recorded on a PVA/GQD film with 0.8%wt GQDs. Figure 7a,b correspond to contact-mode AFM (a) and UFM (b) images simultaneously recorded over the same surface area. The apparently crystalline 3D islands in Figure 7a are characterized by stepped facets with characteristic orientations and angles. Figure 7c corresponds to the derivative of the topography (Figure 7a) and has been included to facilitate the observation of topographic slope variations. 

As in Figure 5c, the topography reveals the formation of 3D islands of crystalline appearance. The fact that thin inorganic layered fillers may induce the crystallization of polymer nanocomposites is already well known [45]. In particular, the formation of PVA crystallites in the presence of sodium montmorillonite (MMT) has been reported [46]. Furthermore, it has also been shown that the introduction of a certain amount of graphene nanosheet fillers enhances PVA crystallinity, this effect being attributed to graphene acting as a nucleating size for PVA crystallization [47]. Therefore, it is quite plausible that GQDs may also induce the formation of crystalline PVA domains. It should be noted that the islands in Figure 5a are much larger than the ~4.2 nm size estimated from the XRD data for the PVA crystalline domains, according to the Debye-Scherrer equation (see discussion related to Figure 2). The fact that XRD on this film does not provide any indication of PVA crystal growth suggests that most probably their formation mainly occurs on the film surface, whereas the XRD information comes not only from the surface, but from the whole PVA film. Furthermore, it could be the case that the specific domains in which the polymer atomic species are sufficiently well-ordered to contribute to the XRD signal are much smaller than the island size. In addition, the structure of the formed PVA crystallites may differ from that of the crystalline domains within the pristine semicrystalline PVA film. As a matter of fact, in [46], the authors concluded that the structure formed next to the MMT surface corresponded to a new crystal structure of PVA. If the intensity of the XRD signal originating from diffraction at the formed crystalline domains is low and the peak is broad, it might well be that it cannot be resolved in our XRD measurements. 

The islands contrast in UFM (Figure 7b) is facet-dependent, and it is probably strongly influenced by the orientation of the facet with respect to the tip. The chemical termination of the facet surface may also play an important role in the tip-sample adhesion, and, thus, in the resulting UFM signal. 

Figure 7d shows the height-contour profile along the line A–B in Figure 7a, according to which the island height reaches ~180 nm; different island facets can be appreciated. 

Next to those apparently crystalline islands, flatter rounded terraces can also be distinguished in Figure 7a, such as those marked with arrows. Figure 7e is a height-contour profile along the line C–D in Figure 7a that crosses one of these terraces. As seen in Figure 7c, the height of this terrace (indicated by a dashed line at the contour-profile curve) is ~58 nm, much higher than the terraces found on the PVA/GQD film with 0.4%wt GQDs (see Figure 6). These terraces are also distinguishable in the UFM image (Figure 7b), but they provide no distinct UFM contrast, apart from that originating from the slope changes at their edges. 

Interestingly, in Figure 7b, areas with a higher (brighter) UFM contrast are noticeable in the images at regions with no straightforwardly correlated topographic features, such as the one labelled as “E”. There is no correlation between the brightest UFM zones in Figure 7b and specific features in Figure 7a,c. Figure 7f–h correspond to topographic and LFM images recorded over the area within the dashed white rectangle in Figure 7b, scanning from right to left (g) and from left to right (h). A comparison of Figure 7b,g,h indicates that the stiffer areas in UFM (brighter contrast) exhibit lower friction (darker in (e) and brighter in (a)). Still, no clear correlation between the LFM images and the surface topography (Figure 7f) is noticeable for this area. This type of contrast may arise from the existence of buried PVA crystallite domains in the near subsurface region, positioned very close to the surface, thereby exerting an influence on the tip-sample frictional response. PVA crystals with stiffer contrast and lower friction formed in the presence of an inorganic filler surface (sodium montmorillonite) have been previously observed using AFM modes [46].

Finally, it should be noticed that the aforementioned circular terraces (marked with arrows in Figure 7), do not show a significant frictional contrast in Figure 7g,h with respect to the substrate. 

The images in Figure 8 were recorded on a PVA/GQD film with 1.2%wt GQDs. Figure 8a,b correspond to simultaneously recorded contact-mode AFM topographic (a) and UFM (b) images; Figure 8c,d are also simultaneously recorded contact-mode AFM topographic (c) and UFM (d) images from another surface area of the same sample. 

As seen in Figure 8a,c and Figure 5d, the incorporation of a higher amount of GQDs (1.2%wt) leads to the formation of circular “pores” (e.g., those marked by continuous white arrows in Figure 8a,c) with various diameters, up to ~500 nm, and a depth of ~4 nm. In addition to the pores, circular structures with a surrounding annular rim are apparent (e.g., those marked by the dashed white arrows in Figure 8a,c). The internal diameter of such structures is approximately the same size as that of the pores, but their central area is higher than that of the substrate, their structure and origin being presumably common to those of the pores, but the latter being filled by additional molecules. In the circular structure, marked by the dashed white arrow in Figure 8a, the rim is formed by clusters ~125 nm in diameter and ~5 nm in height; a cluster similar to those at the rim is also located in the central region.

Figure 8e shows a height-contour profile along the white line in Figure 8c from A to B, crossing one of the circular structures and a pore located nearby. Notice that the depressed central region of the circular structure is ~2 nm higher than the surrounding substrate. 

In UFM (Figure 8b,d), the pores usually appear more rigid (brighter contrast). The passivating nature of the GQDs’ central carbon cores may induce the formation of these pores as GQDs are incorporated into the PVA matrix. At some areas, such as the one enclosed by a dashed white circle in Figure 8a,b, the fact that the UFM image yields a stiffer contrast distinctly suggests that the topography corresponds to a covered pore area. Regarding the circular structure marked by the dashed white arrow in Figure 8c, the UFM (darker) contrast is clearly indicative of a softer zone. We understand this softer contrast has its origin in GQD-modified PVA clusters located both at the edges and filling the central part. 

When analyzing Figure 5 (0.4%wt GQD loading), we observed many clusters that, despite being topographically similar, yielded a lower UFM contrast, indicative of lower stiffness. We also identified defects, which we termed “void” defects, that apparently consisted in displaced PVA surface clusters. We attributed the origin of these clusters to the incorporation of a slight amount of hydrophilic GQDs into the PVA matrix. These GQDs attached to the PVA molecular chains via H bonding, influencing their interactions and conformation. We pointed out that the passivating nature of the GQDs’ central carbon core could play a role in inducing the “voids”. For 1.2%wt GQD loading in Figure 8, we observe “pores” much larger in diameter than the “voids”, which, however, could have a similar origin, but this time, requiring a higher amount of GQDs interacting with each other and with the PVA segments to induce these modifications. 

**Figure 9:**
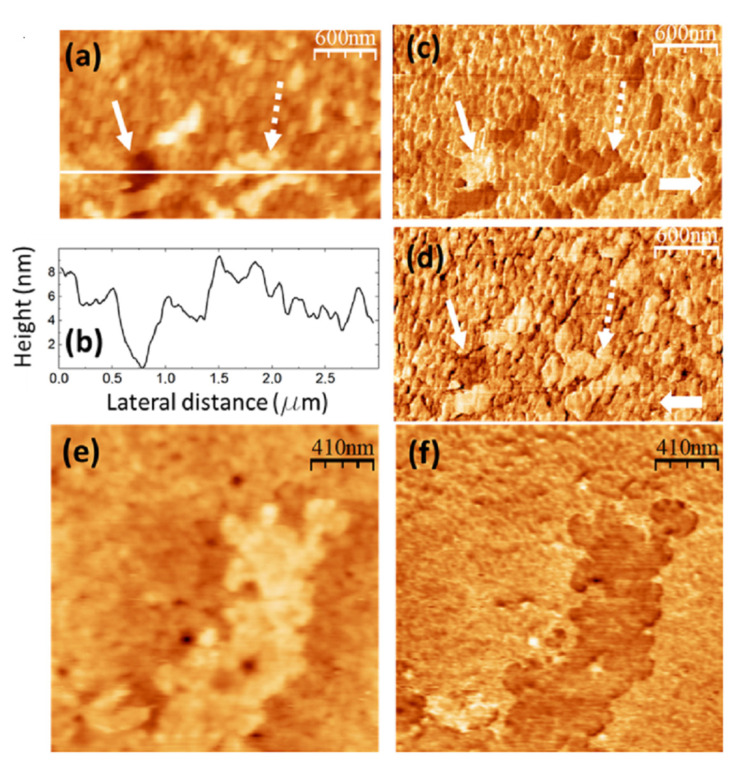
PVA/GQD film with 2.0%wt GQDs. (**a**) Contact-mode AFM topography. Color-scale range: 12 nm. (**b**) Height-contour profile recorded along the lower white line in (**a**). (**c**,**d**) LFM images recorded over the same area as (**a**), scanning from left to right (**c**) and from right to left (**d**). (**e**) Contact-mode AFM topography on a different surface area than (**a**). Color-scale range: 17 nm. (**f**) UFM image recorded simultaneously with (**e**), over the same surface area.

The images in Figure 9 were recorded on a PVA/GQD film with 2.0%wt GQDs. Figure 9a corresponds to the contact-mode AFM topography, Figure 9b is a height-contour profile along the white line in Figure 9a, and Figure 9c,d are LFM images recorded over the same surface area as Figure 9a, scanning from right to left (Figure 9c) and from left to right (Figure 9d). Also, in this film, we find pores similar to those in Figure 8, such as the one marked by the continuous white arrow in Figure 9a, with a diameter of ~500 nm and a depth of ~4 nm (see Figure 9b). At the pore zone, LFM reveals a higher frictional contrast (brighter in Figure 9c and darker in Figure 9d. According to Figure 9a–d, for the 2.0%wt GQD load, the surface is characterized by the presence of cluster aggregates (e.g., the one marked by the dashed white arrow in Figure 9a) that yield a clear lower frictional contrast (Figure 9c,d). 

Figure 9e,f correspond to contact-mode AFM topography and UFM image, simultaneously recorded over the same surface area, different from that of Figure 9a, on the same sample. From the figures, it is noticeable that the aggregates in Figure 9a gather to form extended terraces that yield a distinct softer (darker) UFM contrast, confirming that distinct phases characterized by different elastic and frictional contrast form on the film surface. It should be remarked that for GQD loading higher than 1.2%wt GQD, a GQD-related band emerges in the FT-IR spectrum (see Figure 3). We understand that the new PVA-GQD phase observed in Figure 9 develops as a result of the incorporation of GQDs to the PVA molecular chains via H bonds and the arrangement of the modified PVA molecules in a distinct conformation. This arrangement possibly also involves GQD-GQD interactions. 

**Figure 10:**
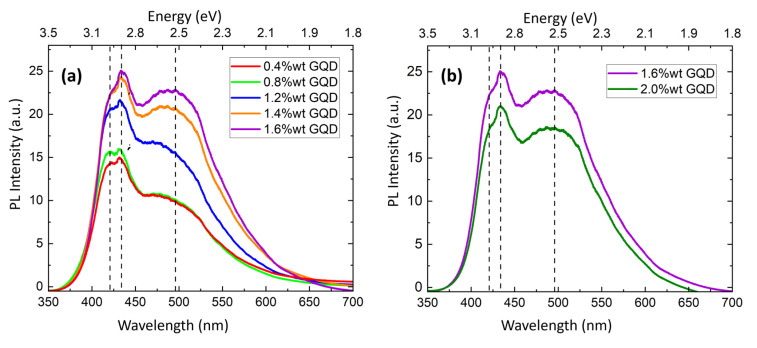
(**a**) Room temperature (RT) PL spectra of PVA/GQD films, obtained under 325 nm wavelength excitation for the set of nanocomposites. (**b**) RT PL spectra of the composites with the two highest GQDs loads.

Figure 10 shows the PL spectra of the PVA/GQDs composite films. In PVA/GQD films, the PL response is due to the incorporation of the GQDs and is excitation-dependent [12,14,16]. Our PL measurements were conducted at room temperature, utilizing a 325 nm laser source. No significant PL emission was detected in pure PVA films. As seen in Figure 10, the PL spectra of the PVA/GQD films exhibit maxima at ~420 nm, ~434 nm, and ~495 nm (marked with dashed lines in Figure 10a,b). The PL emission intensity increases as the concentration of GQDs is increased, although in a nonlinear manner. In addition, the PL curves experience significant variations in shape, reflecting that the relative contribution of each spectral component varies with the amount of GQD incorporated into the PVA matrix. 

To date, there is still a need for a comprehensive understanding of the mechanisms behind the PL emission of GQDs [48,49]. There are three primary contributing factors: size, surface structure, and edge effects. The quantum confinement effect of conjugated p-domains is determined by the carbon core. Surface states are determined by the hybridization of the carbon backbone and the connected chemical groups. Various functional groups (C–OH, C=O, O–C=O, etc.) introduced during the growth of GQDs can give rise to surface states with energy levels located between the p and p* states of C=C, leading to the absorption/emission bands due to electron transitions within one or more of these groups. Both the edge structure and the presence of defects/surface states can significantly alter the electronic properties of GQDs. PL emission of GQDs primarily arises from the interplay between intrinsic state emission and defect state emission. Intrinsic state emission results from the quantum size effect, zigzag edge sites, or the recombination of localized electron-hole pairs, whereas defect state emission originates from energy traps. Ref. [4] provides a typical PL emission spectrum of the commercial GQDs excited at 350 nm. 

Our PVA/GQD films, transparent under natural light, exhibited a bright blue color when placed inside a UV chamber (see Figure 1), even at the lowest considered GQD loads (0.4%wt GQD). An increase in GQD loading from 0.4 to 0.8%wt resulted in the growth of 3D islands on the PVA/GQD film surface (see Figure 5 and Figure 7), but the PL response did not experience significant variations (see Figure 10a). This result suggests that when GQDs act as nucleating agents for the growth of PVA crystallites, their PL response is quenched. However, for the 1.2%wt GQD load, a steady increase of the PL spectral response is observed. As the amount of incorporated GQDs is further increased, the intensity of the PL band at lower energy (495 nm) increases, along with the emergence of the GQD-related band ~1650 cm^−1^ in FT-IR, while the higher energy PL band (maxima at ~420 and ~434 nm) reaches a saturation value. For ~2%wt GQD (Figure 10b), the overall PL spectral response decreases linearly. 

The PL spectra in Figure 10 can be interpreted with the lower energy band (~420–434 nm) primarily arising from the individual GQD peaks (Figure 2 from [4]), whose energy level is only slightly modified due to the interaction with the PVA matrix. The additional broad PL peak at 495 nm that experiences a larger increase as the amount of GQDs is increased in Figure 10a is possibly originating due to interactions between the GQDs within the PVA matrix. GQDs are expected to form H-bonds with the PVA chains; as the amount of GQDs increases, GQDs attached to the same or different chains may induce a different conformation of the PVA chains and/or interact with each other, thus resulting in a modification of their PL emission. The quenching of the PL signal observed in Figure 10b occurs in correlation with the observation by SPM of a new extended phase with a clear and distinct elastic and frictional contrast (see Figure 9). Such a phase can be reasonably assigned to a new characteristic GQD/PVA configuration. 

It’s noteworthy that the PVA/GQD films retain these PL features after being stored under ambient conditions for at least two years. Our results emphasize the impact of the surface molecular rearrangements and morphology on the PL response of PVA/GQD films. Further experiments are planned to explore the PL behavior in higher detail. 

## 4. Summary and Conclusions

Luminescent polyvinyl alcohol (PVA)/graphene quantum dots (GQDs) polymer nanocomposite films were prepared with varying GQD content ranging from 0.4 to 2.0 weight percentage of GQD NPs relative to PVA (%wt) and were characterized using XRD, FT-IR, TGA, DTA, AFM, LFM, UFM, and PL spectroscopy. 

XRD, FT-IR, and DTA collectively indicate that the percentage crystallinity of the PVA film is not modified by the incorporation of GQDs into the matrix. For loads larger than 1.2%wt GQDs, a GQD-related band is observed at ~1650 cm^−1^ in FT-IR. 

According to the TGA results, for the film with 0.4%wt GQDs, the maximum weight loss corresponding to the evaporation of the residual water occurs at about 15 °C lower temperature than in the pure PVA and the other PVA/GQD films. The DTA reveals no significant modification of the films glass transition and melting point temperatures for the different GQD loadings, even though a markedly different degradation behavior is observed at temperatures above the melting point. 

AFM reveals significant modifications of the film surface topography for the different GQDs loading. For 0.4%wt GQD, the topography is characterized by small “void” defects apparently formed by displaced PVA clusters, and surface terraces form, with no significant characteristic frictional or elastic contrast. At a GQD concentration of 0.8%wt, the film surface exhibits the formation of 3D islands with a typically crystalline morphology. This observation strongly indicates that the GQDs are likely serving as nucleating agents for PVA surface island growth. For 1.2%wt GQD, the surface is characterized by “pores” up to ~500 nm in diameter, ~4 nm deep exhibiting higher stiffness and friction contrast. Additionally, circular structures of similar diameter as the pores are rimmed by clusters that yield lower stiffness and friction. For films with 2.0%wt GQD concentration, extended cluster aggregates with distinctly lower friction and stiffness define a new phase on the film surface. 

The PL emission of the PVA/GQD films exhibits maxima at ~420 nm, ~434 nm, and ~495 nm and shows a dependence on both the amount of GQDs incorporated and the film surface morphology. Almost no modification of the PL signal is observed when comparing the films with 0.4%wt GQDs and 0.8%wt GQDs, which suggests that the GQDs involved in promoting the growth of 3D PVA islands do not contribute to the PL signal. The maxima at ~495 nm, corresponding to a broader band, experience a higher increase in intensity compared to the other PL spectral components when the GQD loading is increased above 1.2%wt. The PL emission saturates and diminishes for the films with 2%wt GQD loads, in correlation with the appearance of the new extended surface phase. 

The results revealed in this study provide valuable insights into how the structure and surface morphology of PVA/GQD films influence their photoluminescent (PL) response. This understanding is of paramount importance in advancing and fine-tuning these materials for a wide array of applications, including optoelectronic devices, sensors, and nanophotonic technologies. Additionally, knowledge of the impact of the incorporation of GQDs on the surface molecular rearrangements of PVA/GQD films is essential for comprehending their potential influence and behavior when employed as imaging agents in biomaterials or as drug delivery careers. 

## Figures and Tables

**Table 1:** Peak assignment in the FT-IR spectra in Figure 3.

FT-IR Wavenumber (cm^−1^)	Peak Assignment
3600–3100	O–H (stretching)
2935	CH_2_ (symmetric)
2908	CH_2_ (asymmetric)
1650	absorbed water/GQD
1560	C=O/COOH
1417	–OH (bending)
1327	C–H (wagging)
1141	C–O/C–C (stretching)
1089	C–O (stretching)
916	CH_2_ (rocking)
842	C–C (stretching)

**Table 2:** Weight loss percentage for each degradation step.

Weight Loss %	Stage I	Stage II	Stage III	Stage IV	Remains (500 °C)
Pure PVA	5	61	12	17	5
0.4%wt GQD	5	51	17	18	9
1.2%wt GQD	5	58	15	12	10
1.6%wt GQD	5	58	15	15	7
2.0%wt GQD	5	61	12	15	7

## Data Availability

The data presented in this study are available on request from the corresponding author.
